# A new image encryption scheme based on fractional-order hyperchaotic system and multiple image fusion

**DOI:** 10.1038/s41598-021-94748-7

**Published:** 2021-08-03

**Authors:** Xinyu Gao, Jiawu Yu, Santo Banerjee, Huizhen Yan, Jun Mou

**Affiliations:** 1 School of Information Science and Engineering, Dalian Polytechnic University, Dalian, 116034 China; 2 Department of Mathematical Sciences, Giuseppe Luigi Lagrange, Politecnico di Torino, Corso Duca degli Abruzzi 24, Torino, Italy

**Keywords:** Mathematics and computing, Physics

## Abstract

A multi-image encryption scheme based on the fractional-order hyperchaotic system is designed in this paper. The chaotic characteristics of this system are analyzed by the phase diagram, Lyapunov exponent and bifurcation diagram. According to the analyses results, an interesting image encryption algorithm is proposed. Multiple grayscale images are fused into a color image using different channels. Then, the color image is scrambled and diffused in order to obtain a more secure cipher image. The pixel confusion operation and diffusion operation are assisted by fractional hyperchaotic system. Experimental simulation and test results indicate that the devised multi-image encryption scheme can effectively encrypt multiple images, which increase the efficiency of image encryption and transmission, and have good security performance.

## Introduction

In the era of big data, picture information is widely spread on the network, and the security of picture information is also widely concerned^1^. Conventional encryption schemes such as AES, DES encrypt textual data and do not apply to the encryption of images^2–4^. New image encryption algorithms, especially chaos-based encryption algorithms, are under increasingly investigation. Lorentz discovered chaotic attractors in 1963, and in 1997, Fridrich first applied chaotic systems to digital image encryption^5–8^. Chaotic systems are widely used in image encryption and have become a hot research topic in the field of secure communication because of their sensitivity to initial values and irregular internal random motion in deterministic systems^1,9–16^. Compared with ordinary chaotic systems, hyperchaotic systems have more complex dynamics and greater sensitivity and are more suitable for image encryption^17–22^. The fractional-order chaotic system is also more secure because the key space is increased by adding system variables^2,23–28^. Therefore, in this encryption scheme, the fractional-order hyperchaotic system is used for image encryption.

The prerequisite for employing fractional-order chaotic systems is to be able to solve them out. Commonly used methods for solving fractional order chaotic systems are time domain-frequency domain solution algorithms, prediction-correction algorithms, and Adomian decomposition method(ADM)^29,30^. The ADM is widely used due to the advantages of fast convergence and high solution accuracy. However, in the case of conformable fractional calculus, the conformable ADM (CADM) is needed to obtain the digital solution of the chaotic system^31,32^.

Another noteworthy point is that single-image encryption is fast but inefficient^21^. Multi-image encryption can encrypt two or more images at a time with the same computational complexity, which has increased the effectiveness of image encryption^33–37^. Many multi-image encryption schemes are already proposed by scholars. Combined with nonlinear fractional Merlin transform and discrete cosine transform, Pan et al. proposed an optical multi-image encryption scheme^38^. On this basis, Zhou et al. proposed a dual image encryption algorithm based on co-sparse representation and random pixel exchange^39^. Zhang et al. proposed a multi-image encryption scheme to encrypt the arbitrary number of images^37^ and by using a DNA encoding encryption algorithm to accomplish encrypt multiple images simultaneously^35^. There also some scholars proposed the encryption schemes that can encrypt arbitrary size multiple images or a batch of images^40–43^. Huang et al. proposed a double-image encryption algorithm based on compression- sensing, which reduces data space while improving encryption efficiency^44^. These encryption schemes all use chaotic systems, which greatly improve the randomness of the encrypted image data and make the encryption schemes withstand a certain level of hacking^40,45^. However, some of the encryption schemes still have the problem of weak security or lack of efficiency. For this reason, a new encryption scheme based on fractional-order hyperchaotic systems and multi-image fusion is proposed^46–51^. The application of fractional-order hyperchaotic system makes the pseudo-random sequence more complex and thus allows for a more secure encryption algorithm^52^. The fusion of multiple images allows image encryption efficiency to be improved.

The remaining part of the paper is arranged as the following. "Characteristic analysis of a fractional-order hyperchaotic system" section, the circuit and the dynamic analysis of chaotic system are given. The encryption algorithm which includes scrambling and diffusion is shown in "The complete encryption scheme" section. "Decryption scheme" section introduces the complete encryption and decryption scheme. "Performance analysis" section illustrates the simulation results and some security analyses. In the last section "Conclusion", this paper ends with concluding remarks.

## Characteristic analysis of a fractional-order hyperchaotic system

### Fractional-order memristive hyperchaotic circuit

**Figure 1:**
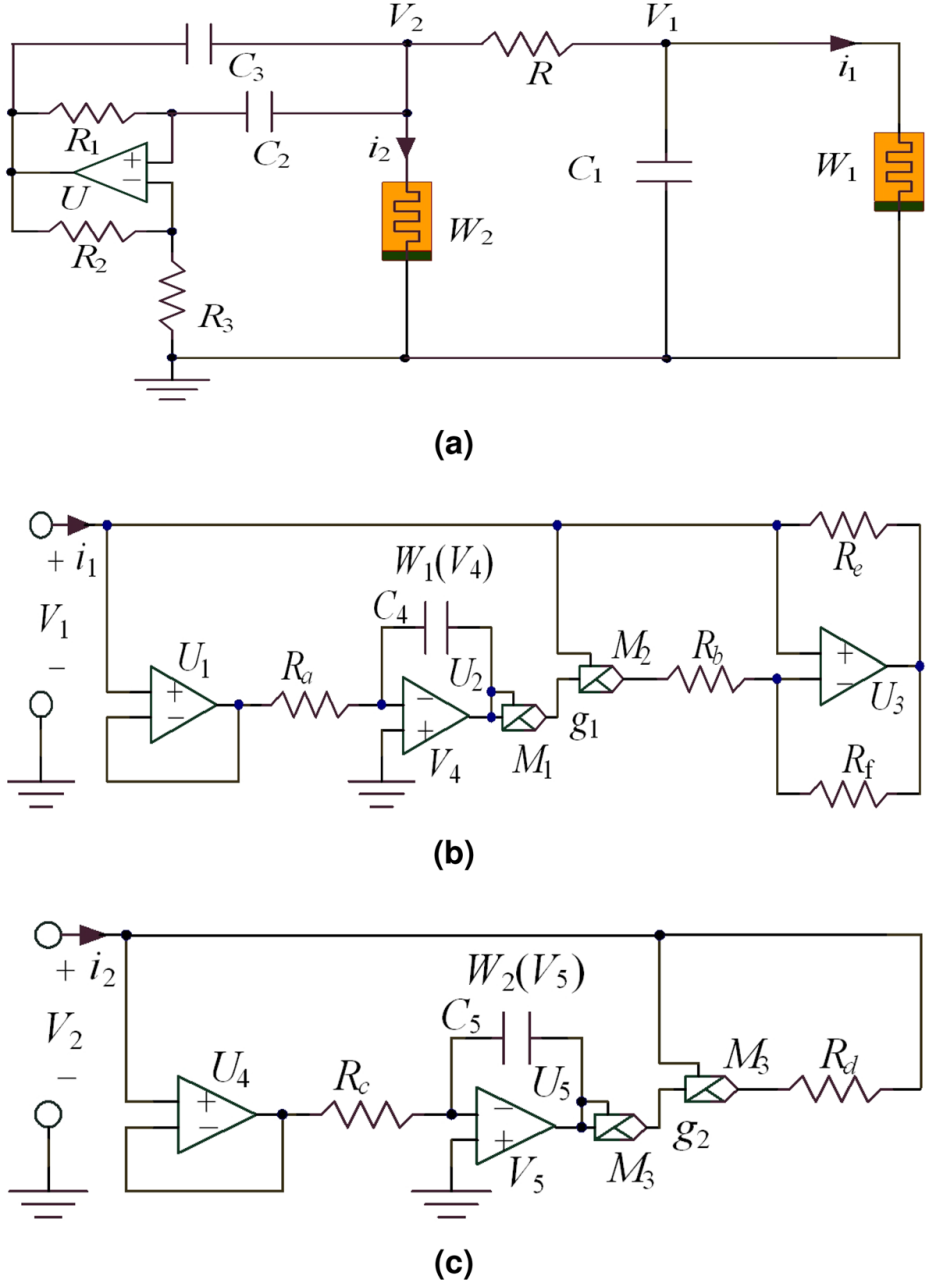
Memristive circuit, (**a**) BPF memristive Chua’s circuit, (**b**) equivalent circuit for the memristor *W*$_1$, (**c**) equivalent circuit for the memristor *W*$_2$.

A new two-memristor circuit based on band pass filter (BPF) and Chua’s circuit is obtained as shown in Fig. 1a. The two equivalent circuits of two memristors *W*$_1$ and *W*$_2$ are shown in Fig. 1b, c.

For the Fig. 1b, *V*$_1$ and *i*$_1$ represent the input voltage and the input current, *V*$_4$ is the node voltage of the integrator *U*$_2$ output. Therefore, the memristor *W*$_1$ can be expressed as

1 $$\begin{aligned} {\left\{ \begin{array}{ll} {i_1}={W_1}{(V_4)}{V_1}=-\frac{1}{{R_b}}(1-m_1{V_4}^2){V_1}\\ \frac{d{V_4}}{dt}=f({V_1})=-\frac{1}{{R_a}{C_4}}\\ \end{array}\right. } \end{aligned}$$

where, *m*$_1$ represent the total gain of multipliers *M*$_1$ and *M*$_2$. The flux$\phi _1$(t) of the memristor *W*$_1$ is

2 $$\begin{aligned} \varphi _1({t})=\int _{-\infty }^{{t}}{V_1}(\tau )d(\tau )=-{R_a}{C_4}{V_4}({t}) \end{aligned}$$

For the Fig. 1c, *V*$_2$ and *i*$_2$ represent the input voltage and the input current, *V*$_5$ means that the node voltage of the integrator *U*$_5$ output. Therefore, the memristor *W*$_2$ is expressed as

3 $$\begin{aligned} {\left\{ \begin{array}{ll} {i_2}={W_2}{(V_5)}{V_2}=-\frac{1}{{R_d}}(1-m_2{V_5}^2){V_2}\\ \frac{d{V_5}}{dt}=f({V_2})=-\frac{1}{{R_c}{C_5}}\\ \end{array}\right. } \end{aligned}$$

where, *m*$_2$ represent the total gain of multipliers *M*$_3$ and*M*$_4$. The flux$\phi _2$(t) of the memristor *W*$_2$ is

4 $$\begin{aligned} \varphi _2({t})=\int _{-\infty }^{{t}}{V_2}(\tau )d(\tau )=-{R_c}{C_5}{V_5}({t}) \end{aligned}$$

### Chaotic system

According to the Kirchhoff’s circuit laws, current-voltage relation of capacitors and memristor model, we get the they mathematical model is

5 $$\begin{aligned} {\left\{ \begin{array}{ll} \frac{d{V_1}}{dt}=-\frac{1}{{R}{C_1}}({V_1}-{V_2})+\frac{1}{{R_b}{C_1}}(1-m_1{V_4}^2){V_1}\\ \frac{d{V_2}}{dt}=-\frac{1}{{R}{C_2}}({V_1}-{V_2})+\frac{1}{{R_d}{C_1}}(1-m_2{V_5}^2){V_2}-\frac{2s+1}{(s+1){R_1}{C_2}}{V_3}\\ \frac{d{V_3}}{dt}=-\frac{s+1}{{R}{C_3}}({V_1}-{V_2})+\frac{s+1}{{R_d}{C_3}}(1-m_2{V_5}^2){V_2}-\frac{2}{{R_1}{C_3}}{V_3}\\ \frac{d{V_4}}{dt}=-\frac{1}{{R_a}{C_4}}{V_1}\\ \frac{d{V_5}}{dt}=-\frac{1}{{R_c}{C_5}}{V_2}\\ \end{array}\right. } \end{aligned}$$

where, *s* = *R*$$_3$$/*R*$$_2$$.

For the Eq. (5), introducing the new variables and scaling the circuit parameters as

6 $$\begin{aligned} {\left\{ \begin{array}{ll} x={V_1},y={V_2},z={V_3},w={V_4},u={V_5}\\ C={C_2}={C_3},{R_a}{C_4}={R_c}{C_5}\\ c=\frac{{C}}{{C_1}},e=\frac{{R}{C}}{{R_b}{C_1}},g=\frac{{R}}{{R_d}},n=\frac{{R}}{{R_1}},p=\frac{{R}{C}}{{R_a}{C_4}}\\ \end{array}\right. } \end{aligned}$$

According to Eq. (6), the Eq. (5) becomes to

7 $$\begin{aligned} {\left\{ \begin{array}{ll} \dot{x}=-c(x-y)+e(1-m_1w^2)x\\ \dot{y}=-s(x-y)+sg(1-m_2u^2)y-(2s+1)/(s+1)nz\\ \dot{z}=-(s+1)(x-y)+(s+1)g(1-m_2u^2)y-2nz\\ \dot{w}=-px\\ \dot{u}=-py\\ \end{array}\right. } \end{aligned}$$

Based on Eq. (7), the fractional-order memristive hyperchaotic circuit system is denoted by

8 $$\begin{aligned} {\left\{ \begin{array}{ll} ^*D_{t_0}^qx=-c(x-y)+e(1-m_1w^2)x\\ ^*D_{t_0}^qy=-s(x-y)+sg(1-m_2u^2)y-(2s+1)/(s+1)nz\\ ^*D_{t_0}^qz=-(s+1)(x-y)+(s+1)g(1-m_2u^2)y-2nz\\ ^*D_{t_0}^qw=-px\\ ^*D_{t_0}^qu=-py\\ \end{array}\right. } \end{aligned}$$

where, *q* is order of the equation.

According to the CADM^43^ algorithm, the linear and nonlinear terms of the fractional-order system are obtained as follows

9 $$\begin{aligned} \begin{bmatrix} Lx\\ Ly\\ Lz\\ Lw\\ Lu\\ \end{bmatrix} = \begin{bmatrix} (e-c)x+cy\\ -sx+sy(g+1)-(2s+1)/(s+1)nz\\ -(s+1)x+(s+1)y(g+1)-2nz\\ -px\\ -py\\ \end{bmatrix} , \begin{bmatrix} Nx\\ Ny\\ Nz\\ Nw\\ Nu\\ \end{bmatrix} = \begin{bmatrix} -em_1w^2x\\ -sgm_2u^2y\\ -(s+1)gm_2u^2y\\ 0\\ 0\\ \end{bmatrix} \end{aligned}$$

The before five Adomian polynomials for the nonlinear parts *-cm*$_1$ *w* $_2$, *-sgm*$_2$, *-u*$_2$ and -(*s*+1)*gm*$_2$ *u*$_2$ are

10 $$\begin{aligned}&{\left\{ \begin{array}{ll} A_{-cm_1x(w)^2}^0=&{}-cm_1x^0(w^0)^2\\ A_{-cm_1x(w)^2}^1=&{}-cm_1x^1(w^0)^2-2cm_1x^1w^1w^0\\ A_{-cm_1x(w)^2}^2=&{}-cm_1x^2(w^0)^2-2cm_1x^1w^1w^0-2cm_1x^1w^2w^0-cm_1x^0(w^1)^2\\ A_{-cm_1x(w)^2}^3=&{}-cm_1x^3(w^0)^2-2cm_1x^2w^1w^0-2cm_1x^0w^3w^0-2cm_1x^0w^2w^1\\ {} &{}-2cm_1x^1w^2w^0-cm_1x^1(w^1)^2\\ A_{-cm_1x(w)^2}^4=&{}-cm_1x^4(w^0)^2-2cm_1x^3w^1w^0-2cm_1x^2w^0w^2-2cm_1x^1w^2w^1\\ {} &{}-2cm_1x^1w^3w^0-2cm_1x^0w^4w^0-2cm_1x^0w^3w^0-2cm_1x^2(w^1)^2\\ {} &{}-2cm_1x^0(w^2)^2\\ \end{array}\right. } \end{aligned}$$

11 $$\begin{aligned}&{\left\{ \begin{array}{ll} A_{-sgm_2y(u)^2}^0=&{}-sgm_2y^0(u^0)^2\\ A_{-sgm_2y(u)^2}^1=&{}-sgm_2y^1(u^0)^2-2sgm_2y^1u^1u^0\\ A_{-sgm_2y(u)^2}^2=&{}-sgm_2y^2(u^0)^2-2sgm_2y^1u^1u^0-2sgm_2y^1u^2u^0-sgm_2y^0(u^1)^2\\ A_{-sgm_2y(u)^2}^3=&{}-sgm_2y^3(u^0)^2-2sgm_2y^2u^0u^1-2sgm_2y^0u^3u^0-2sgm_2y^0u^2u^1\\ {} &{}-2sgm_2y^1u^2u^0-sgm_2y^1(u^1)^2\\ A_{-sgm_2y(u)^2}^4=&{}-sgm_2y^4(u^0)^2-2sgm_2y^3u^0u^1-2sgm_2y^2u^0u^2-2sgm_2y^1u^2u^1\\ {} &{}-2sgm_2y^1u^3u^0-2sgm_2y^0u^4u^0-2sgm_2y^0u^3u^0-2sgm_2y^2(u^1)^2\\ {} &{}-sgm_2y^0(u^2)^2\\ \end{array}\right. } \end{aligned}$$

12 $$\begin{aligned}&{\left\{ \begin{array}{ll} A_{-(s+1)gm_2y(u)^2}^0=&{}-(s+1)gm_2y^0(u^0)^2\\ A_{-(s+1)gm_2y(u)^2}^1=&{}-(s+1)gm_2y^1(u^0)^2-2(s+1)gm_2y^1u^1u^0\\ A_{-(s+1)gm_2y(u)^2}^2=&{}-(s+1)gm_2y^2(u^0)^2-2(s+1)gm_2y^1u^1u^0-2(s+1)gm_2y^1u^2u^0-(s+1)gm_2y^0(u^1)^2\\ A_{-(s+1)gm_2y(u)^2}^3=&{}-(s+1)gm_2y^3(u^0)^2-2(s+1)gm_2y^2u^0u^1-2(s+1)gm_2y^0u^3u^0-2(s+1)gm_2y^0u^2u^1\\ {} &{}-2(s+1)gm_2y^1u^2u^0-(s+1)gm_2y^1(u^1)^2\\ A_{-(s+1)gm_2y(u)^2}^4=&{}-(s+1)gm_2y^4(u^0)^2-2(s+1)gm_2y^3u^0u^1-2(s+1)gm_2y^2u^0u^2-2(s+1)gm_2y^1u^2u^1\\ {} &{}-2(s+1)gm_2y^1u^3u^0-2(s+1)gm_2y^0u^4u^0-2(s+1)gm_2y^0u^3u^0-2(s+1)gm_2y^2(u^1)^2\\ {} &{}-(s+1)gm_2y^0(u^2)^2\\ \end{array}\right. } \end{aligned}$$

If the initial conditions are set as *x*0, *y*0, *z*0, *w*0, *u*0, then the first term is

13 $$\begin{aligned} {\left\{ \begin{array}{ll} x^0=x(t_0)\\ y^0=y(t_0)\\ z^0=w(t_0)\\ w^0=w(t_0)\\ u^0=u(t_0)\\ \end{array}\right. } \end{aligned}$$

Let

14 $$\begin{aligned} {\left\{ \begin{array}{ll} c^0_1=x^0\\ c^0_2=y^0\\ c^0_3=w^0\\ c^0_4=w^0\\ c^0_5=u^0\\ \end{array}\right. } \end{aligned}$$

We can get the coefficients of other term as follows

15 $$\begin{aligned}&{\left\{ \begin{array}{ll} c_1^1=&{}-c(c_1^0-c_2^0)+ec_1^0-em_1(c_1^0(c_4^0)^2)\\ c_2^1=&{}-s(c_1^0-c_2^0)+sgc_2^0-scm_2(c_2^0(c_5^0)^2)-\frac{2s+1}{s+1}nc_3^0\\ c_3^1=&{}-(s+1)(c_1^0-c_2^0)+(s+1)gc_2^0-(s+1)gm_2(c_2^0(c_5^0)^2)-2nc_3^0\\ c_4^1=&{}-pc_1^0\\ c_5^1=&{}-pc_2^0\\ \end{array}\right. } \end{aligned}$$

16 $$\begin{aligned}&{\left\{ \begin{array}{ll} c_1^2=&{}-c(c_1^1-c_2^1)+ec_1^1-em_1(c_1^1(c_4^0)^2+2c^0_1c^1_4c_4^0)\\ c_2^2=&{}-s(c_1^1-c_2^1)+sgc_2^1-scm_2(c_2^1(c_5^0)^2+2c_2^0c_5^1c_5^0)-\frac{2s+1}{s+1}nc_3^1\\ c_3^2=&{}-(s+1)(c_1^1-c_2^1)+(s+1)gc_2^1-(s+1)gm_2(c_2^1(c_5^0)^2+2c_2^0c_5^1c_5^0)-2nc_3^1\\ c_4^2=&{}-pc_1^1\\ c_5^2=&{}-pc_2^1\\ \end{array}\right. } \end{aligned}$$

17 $$\begin{aligned}&{\left\{ \begin{array}{ll} c_1^3=&{}-c(c_1^2-c_2^2)+ec_1^2-em_1(c_1^2(c_4^0)^2)-em_1(4c_1^1c_4^1c_4^0+2c^0_1c^2_4c_4^0+4c_4^0(c_4^1)^2)\\ c_2^3=&{}-s(c_1^2-c_2^2)+sgc_2^2-scm_2(c_2^2(c_5^0)^2)-sgm_2(4c_2^1c_5^1c_5^0+4c_2^0(c_5^1)^2)-\frac{2s+1}{s+1}nc_3^2\\ c_3^3=&{}-(s+1)(c_1^2-c_2^2)+(s+1)gc_2^2-(s+1)gm_2(c_2^2(c_5^0)^2)-(s+1)gm_2-(4c_2^1c_5^1c_5^0\\ {} &{}+4c_2^0(c_5^1)^2+2c_2^0c_5^2c_5^0)2nc_3^2\\ c_4^3=&{}-pc_1^2\\ c_5^3=&{}-pc_2^2\\ \end{array}\right. } \end{aligned}$$

18 $$\begin{aligned}&{\left\{ \begin{array}{ll} c_1^4=&{}-c(c_1^3-c_2^3)+ec_1^3-em_1(c_1^3(c_4^0)^2+6c_1^2c_4^0c_4^1)\\ {} &{}-em_1(6(c_1^1c_4^2c_4^0+c_1^0c_4^1c_4^2-c_1^1(c_4^1)^2)-2c_1^0c_4^3c_4^0)\\ c_2^4=&{}-s(c_1^3-c_2^3)+sgc_2^3-scm_2(c_2^3(c_5^0)^2+6c_2^2c_5^0c_5^1)\\ {} &{}-sgm_2(6(c_2^1c_5^2c_5^0+c_2^0c_5^1c_5^2-2c_2^0c_5^3c_5^0)-2c_2^0c_5^3c_5^0)-\frac{2s+1}{s+1}nc_3^3\\ c_3^4=&{}-(s+1)(c_1^3-c_2^3)+(s+1)gc_2^3-(s+1)gm_2(c_2^3(c_5^0)^2+6c_2^2c_5^0c_5^1)\\ {} &{}-(s+1)gm_2(6(c_2^1c_5^2c_5^0+c _2^0c_5^1c_5^2-c_2^1(c_5^1)^2)+2c_2^0c_5^3c_5^0)-2nc_3^3\\ c_4^4=&{}-pc_1^3\\ c_5^4=&{}-pc_2^3\\ \end{array}\right. } \end{aligned}$$

The CADM solution of the fractional-order memristive hyperchaotic circuit system is

19 $$\begin{aligned} x_j({t})= \sum _{i=0}^4c^i_j\frac{(t-t_0)^iq}{i!q^i} \end{aligned}$$

where *j* = 1, 2, 3, 4, 5.

**Figure 2:**
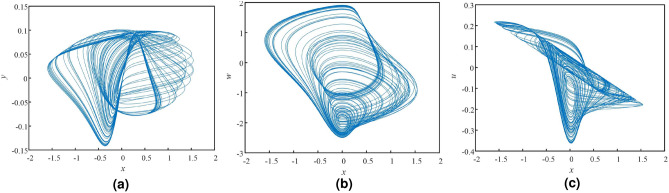
Phase diagrams of fractional-order hyperchaotic system, (**a**) *x*–*y* plan, (**b**) *x*–*w* plan, (**c**) *x*–*u* plan.

**Figure 3:**
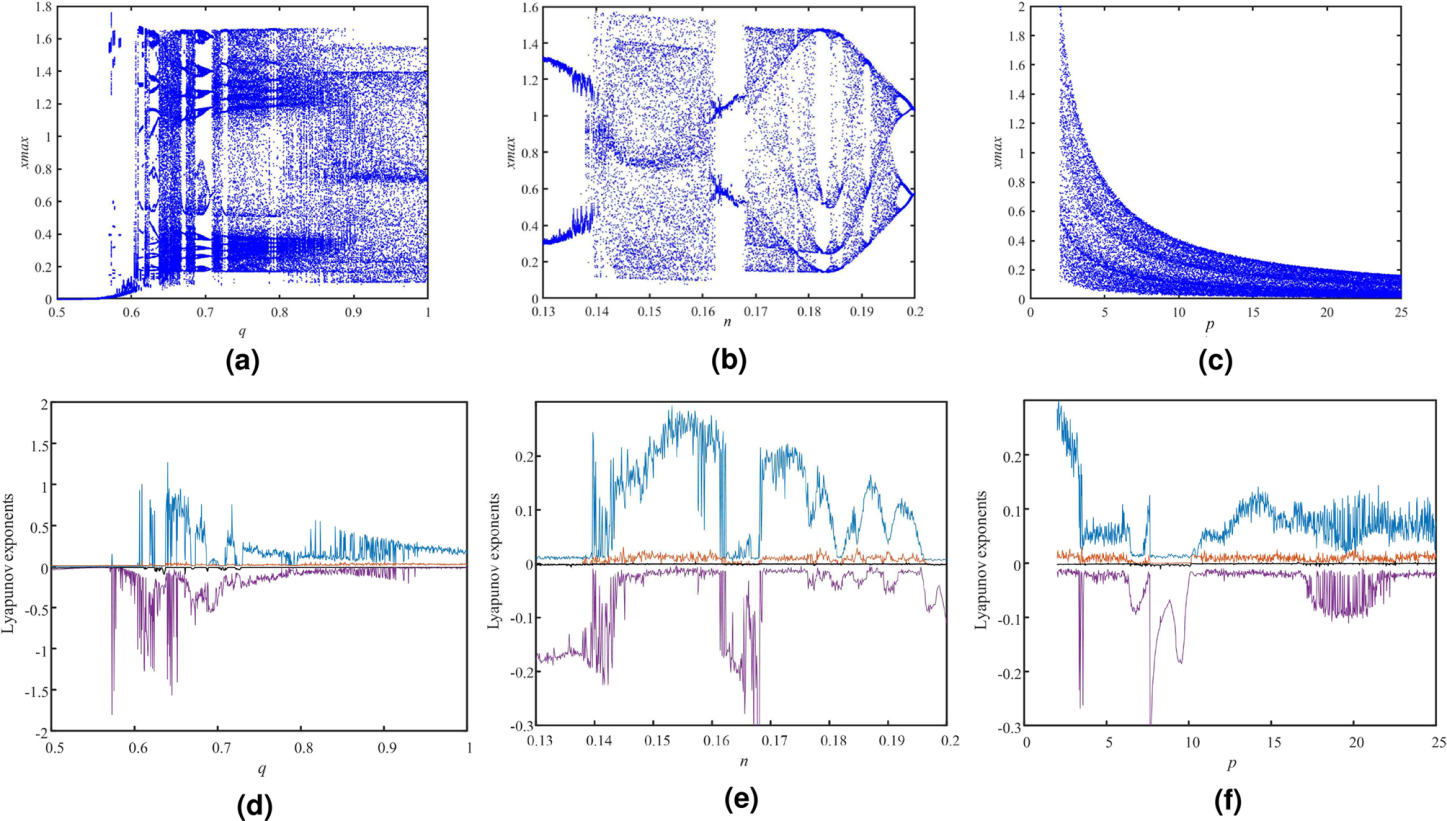
Bifurcation diagrams and Lyapunov exponent spectrums, (**a**) BD, *q* $\in$ (0.5, 1), (**b**) BD, *n* $\in$ (0.13, 0.2), (**c**) BD, *p* $\in$ (2, 25), (**d**) LES, *q* $\in$ (0.5, 1), (**e**) LES, *n* $\in$ (0.13, 0.2), (**f**) LES, *p* $\in$ (2, 25).

Deploying step size *h* = 0.01, *c* = 20, *e* = 150/7, *g* = 15, *n* = 0.15, *p* = 3, *s* = 0.05, *m*$_1$ = *m*$_2$ = 0.1, *q* = 0.97, the starting value are [*x* *y* *z* *w* *u*] = [0.1 0 0 0 0] for the Eq. (8), the phase diagrams with different planes are shown in Figure 2. The attractor trajectories of the fractional-order hyperchaotic system are distributed over a wide area. The bifurcation diagrams (BDs) andu Lyapunov exponent spectrums (LES) are presented in Figure 3 so that we can study the sensitivity of the system with the varying parameter. We severally fix *q* $\in$ (0.5, 1), *n* $\in$ (0.13, 0.2), *p* $\in$ (2, 25) and other parameters are set as above. The fifth Lyapunov exponent is not shown in Fig. 3d–f, because it is much less than 0. From Fig. 3d, when *q* $\in$ (0.5, 0.61), there is no Lyapunov exponent greater than 0. With the increase of *q*, the Lyapunov exponent greater than 0 appears, and the system appears chaotic state. In between there are alternating periodic states and chaotic states appearing. When *n* $\in$ (0.13, 0.2) and *p* $\in$ (2, 25), the changes of Lyapunov exponent spectrum and bifurcation diagram are also consistent. It can be known that the dynamical characteristics of the fractional-order chaotic system is variegated so that the proposed chaotic system is suitable for cryptosystem.

### Equilibrium stability

Qualitative analysis is an effective method to analyze chaos, and the calculation and analysis of the equilibrium point of chaotic system is an important part of the qualitative analysis of chaos mechanism. Continuous fractional-order system is used, so it is essential to find the equilibrium point of the corresponding integer order system to analyze its stability, and then deduce from the integer order to the fractional order. The solution of the differential equation gives the equilibrium point O(*x*$^0_{(e)}$, *y*$^0_{(e)}$, *z*$^0_{(e)}$, *w*$^0_{(e)}$, *u*$^0_{(e)}$) = [0, 0, 0, $\alpha$, $\beta$], and $\alpha$ and $\beta$ on behalf of arbitrary value. For the sake of analysis, if $\alpha$ = 1 and $\beta$ = 1, then the equilibrium point O$_1$(*x*$^0_{(e)}$, *y*$^0_{(e)}$, *z*$^0_{(e)}$, *w*$^0_{(e)}$, *u*$^0_{(e)}$) = [0, 0, 0, 1, 1]. Other system parameters are set in accordance with "Chaotic system" section, and the Jacobian matrix J and its corresponding characteristic equation and eigenvalue can be obtained as follows:

20 $$ J=\left[ {\begin{array}{*{20}c} -0.7143&20.0000&0&0&0\\ -0.0500&0.7250&-0.1571&0&0\\ -1.0500&15.2250&-0.3000&0&0\\ -3.0000&0&0&0&0\\ 0&-3.0000&0&0&0\\ \end{array} } \right] $$

21 $$\begin{aligned}&\lambda ^2(\lambda ^3+0.2893\lambda ^2+3\lambda -1.4464)=0 \end{aligned}$$

22 $$\begin{aligned}&\lambda _1=0,\lambda _2=0,\lambda _3=-0.3703+1.7517i,\lambda _4=-0.3703-1.7517i,\lambda _5=0.4512 \end{aligned}$$

therefore, this equilibrium point is the saddle coke equilibrium point of index 1. Homoclinic and heteroclinic orbits can be formed between saddle points or saddle focal points, which is the key to chaos.

According to the fractional order stability theorem, the system is stable when the system order q satisfies Eq. (23), and it is unstable when the system order q satisfies Eq. (24). Because of Eq. (25), when *q* $\in$ (0.8764, 1), the system is not stable.

23 $$\begin{aligned}&0\le q \le \min _{i=1,2,\ldots ,5}\mid arg(\lambda _i) \mid \end{aligned}$$

24 $$\begin{aligned}&\frac{2}{\pi }\min _{i=1,2,\ldots ,5}\mid arg(\lambda _i) \mid \le q \le 1 \end{aligned}$$

25 $$\begin{aligned}&\mid arg(\lambda _3,4) \mid =1.3625 \end{aligned}$$

### Implementation of DSP technology

**Figure 4:**
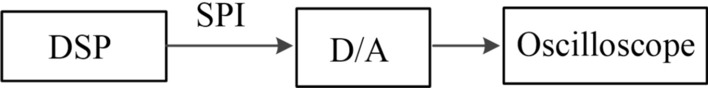
Hardware connection diagram.

**Figure 5:**
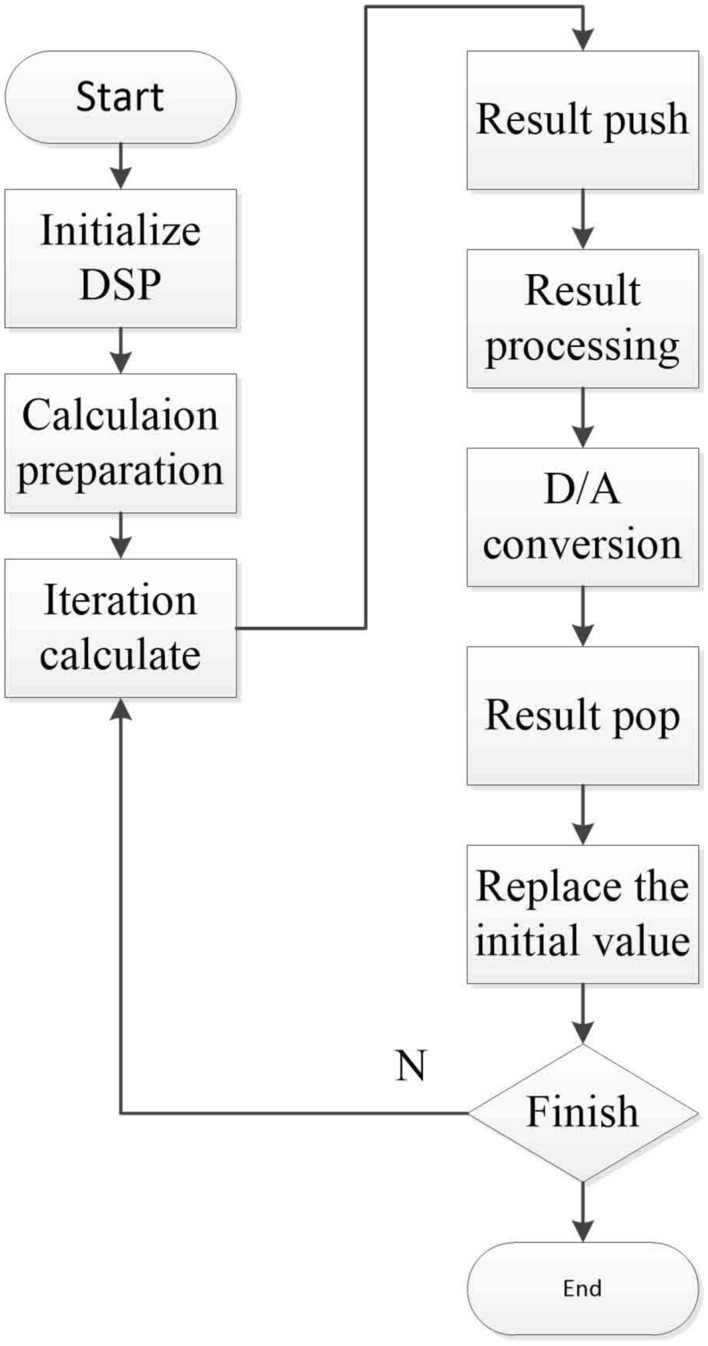
Program flow diagram.

**Figure 6:**
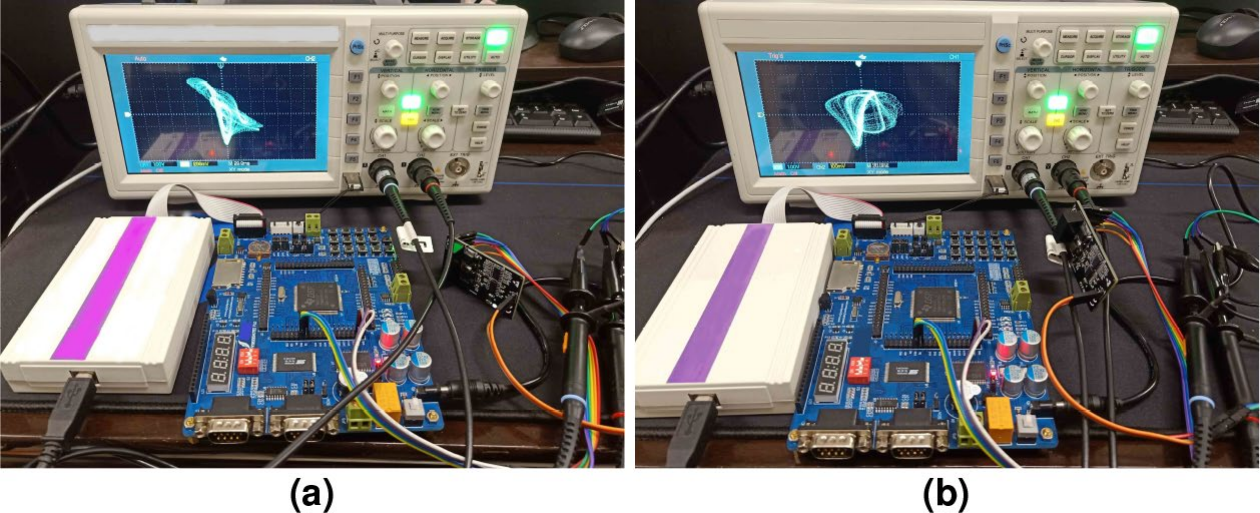
DSP experimental platform construction diagram.

**Figure 7:**
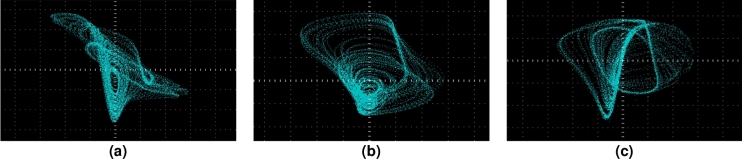
The phase diagrams captured by oscilloscope, (**a**) *x*–*y* plan, (**b**) *x*–*w* plan, (**c**) *x*–*u* plan.

The hardware realization of chaos system can show the possibility of applying chaos from theory to practice. Therefore, DSP experimental platform is built. Through SPI connected to the D/A converter, the final output sequence displayed by the oscilloscope. Hardware connection diagram, program flow diagram and experimental platform construction diagram are shown in the Figs. 4, 5 and 6. Parameter configuration is shown in Table 1. The chaotic phase diagram collected in the oscilloscope is shown in Fig. 7. The output of the oscilloscope is visually consistent with the Fig. 2. This shows that the fractional-order system used can be successfully built on the DSP experimental platform.

**Table 1:** Parameter configuration.

System parameter	*c, e, g, n, p, s, m*$_1$, m$_2$	20, 150/7, 15, 0.15, 3, 0.05, 0.1, 0.1
System initial value	*x, y, z, w, u*	0.1, 0, 0, 0, 0
Order	*q*	0.97
Iteration step size	*h*	0.01

## The complete encryption scheme

**Figure 8:**
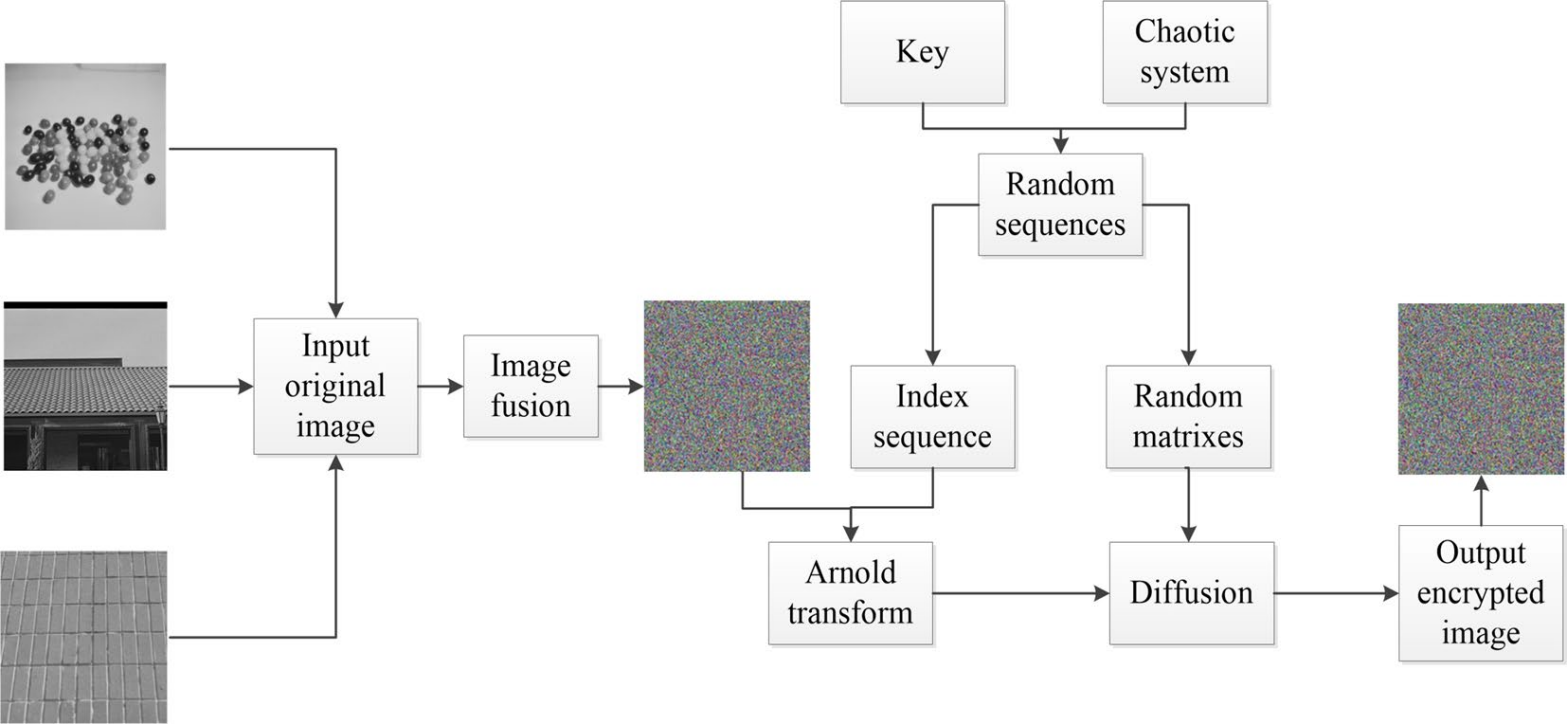
Encryption scheme.

The images combine encryption algorithm based on the principle of color image channels. This is the main discussion point of this section. The process of the proposed encryption scheme is shown in Fig. 8. Firstly, three pictures need to be pre-processed. And then, the pictures are merged and encrypted. Finally, the cipher image is acquired by the image is rotated 180 degrees. The detailed process is described in the following.

### Image fusion

In the step of image fusion, the encrypted gray image can be processed into a color image. The processed image is already visually meaningless.

Step 1: Control parameters and initial values of fractional-order hyperchaotic system are immobilized. The iteration time can be ascertained according to the need.

Step 2: The chaotic sequences *X*, *Y*, *Z*,*W*, *U* can be got from the fractional-order hyperchaotic system based on the Eq (8). The five chaotic sequences are pseudo-random. Simultaneous quantitative operations are performed.

Step 3: Read in three pictures and deal them with bitwise exclusive-OR operation. The bitwise exclusive-OR method is:

26 $$\begin{aligned} {\left\{ \begin{array}{ll} I1=I1\oplus I2\oplus X \\ I2=I2\oplus I3\oplus Y \\ I3=I3\oplus Z \\ \end{array}\right. } \end{aligned}$$

Step 4: Merge three images into one colorful image according to the principles of R, G and B.

Step 5: Finally, the resulting output image I3 is used as the input image for the scrambling operation.

### Scrambling algorithm

Arnold transform is a frequently-used method to scramble the location of the pixels. The process of Arnold transformation is depicted as the following.

Step 1: It is the same as step one and step two of the scrambling algorithm in "Image fusion" section.

Step 2: Two sequences *a*$_1$ and *b*$_1$ are acquired from quantized random sequences. From this, index sequence *q* is generated by addition and modulus through the use of *a*$_1$ and *b*$_1$.

27 $$\begin{aligned} {\left\{ \begin{array}{ll} a_1=X(30000+1:30000+M\times H)\\ b_1=Y(30000+1:30000+M\times H)\\ q=(b_1+a_1.\times (1:M\times H))\%(M\times H)+1 \\ \end{array}\right. } \end{aligned}$$

*M* and *H* are length and width of the original images and (*b*$_1$+*a*$_1$.(1:*MH*))$\%$(*MH*)) means that chaotic sequence a$_1$ is multiplied by the corresponding increasing sequence 1 to *MH*,then add it to b$_1$, and finally take the remainder for *MH*.

Step 3: Every pixel of each of the three images went through. After that, using index sequence can get a rough-and-tumble image by scrambling severally.

Step 4: Three vectors of three images pixels can be got and shaped into matrixes.

### Diffusion algorithm

The operation that the pixels position of an image is unchanged and the pixels values are changed is called diffusion. Idiographic diffusion algorithm processes are as follows.

Step 1: It is the same as step one and step two of the scrambling algorithm in "Image fusion" section.

Step 2: The scrambled image is reused as the source image. The pixel which is located (1, 1) is disposed of.

28 $$\begin{aligned} {\left\{ \begin{array}{ll} C1(1,1)=A1(1,1)\oplus X(1,1)\oplus U(1,1) \\ C2(1,1)=A2(1,1)\oplus X(1,1)\oplus U(1,1) \\ C3(1,1)=A3(1,1)\oplus X(1,1)\oplus U(1,1) \\ \end{array}\right. } \end{aligned}$$

where *A*1$\oplus$*X* is the operation of bitwise exclusive-OR between *A*1 and *X*. *A*1 on behalf of the first scrambled image, *C*1 represents the image which has been diffused. In addition, *A*2, *A*3, *C*2, *C*3 are corresponding with the second image and third image severally.

Step 3: The first row of per image is diffused by

29 $$\begin{aligned} {\left\{ \begin{array}{ll} C1(1,j)=A1(1,j)\oplus X(1,j)\oplus C1(1,j-1) \\ C2(1,j)=A2(1,j)\oplus X(1,j)\oplus C2(1,j-1) \\ C3(1,j)=A3(1,j)\oplus X(1,j)\oplus C3(1,j-1) \\ \end{array}\right. } \end{aligned}$$

where *j* is the number of columns from 2 to end.

Step 4: The first column of per image is diffused by

30 $$\begin{aligned} {\left\{ \begin{array}{ll} C1(i,1)=A1(i,1)\oplus X(i,1)\oplus C1(i-1,1) \\ C2(i,1)=A2(i,1)\oplus X(i,1)\oplus C2(i-1,1) \\ C2(i,1)=A3(i,1)\oplus X(i,1)\oplus C3(i-1,1) \\ \end{array}\right. } \end{aligned}$$

where *i* is the number of rows from 2 to end.

Step 5: For the rest of the pixels, operate on them in a row by

31 $$\begin{aligned} {\left\{ \begin{array}{ll} C1(i,j)=A1(i,j)\oplus X(i,j)\oplus C1(i-1,j)\oplus C1(i,j-1) \\ C2(i,j)=A2(i,j)\oplus X(i,j)\oplus C2(i-1,j)\oplus C2(i,j-1) \\ C2(i,j)=A3(i,j)\oplus X(i,j)\oplus C3(i-1,j)\oplus C3(i,j-1) \\ \end{array}\right. } \end{aligned}$$

three images which are diffused can be obtained.

Step 6: The image which is diffused is rotated 180 degrees.

## Decryption scheme

**Figure 9:**
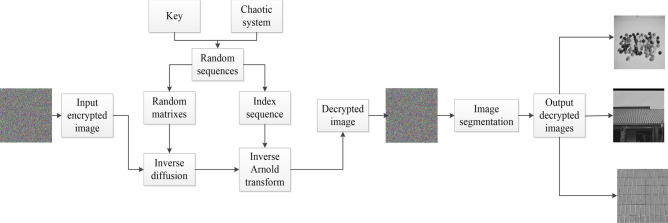
Decryption scheme.

The algorithm for decryption is the reverse operation of the encryption algorithm, the corresponding flowchart is shown in Figure 9. The decryption result is that we can get three undamaged pictures. The detailed algorithm comprises inverse diffusion, inverse Arnold transform and picture segmentation. Some detailed steps are described as follows.

Step 1: As described in step one to two of scrambling algorithm "Image fusion", there are five quantized sequences.

Step 2: The encrypted image is separated into three gray images. Rotate three images 180 degrees, respectively.

Step 3: According to the following Eq. (32)

32 $$\begin{aligned} {\left\{ \begin{array}{ll} D1(1,1)=C1(1,1)\oplus X(1,1)\oplus U(1,1) \\ D2(1,1)=C2(1,1)\oplus X(1,1)\oplus U(1,1) \\ D3(1,1)=C3(1,1)\oplus X(1,1)\oplus U(1,1) \\ \end{array}\right. } \end{aligned}$$

where *C* and *D* represent cipher image and inverse diffused image.

Step 4: The first row of the three figures is treated with inverse diffusion.

33 $$\begin{aligned} {\left\{ \begin{array}{ll} D1(1,j)=C1(1,j)\oplus X(1,j)\oplus C1(1,j-1) \\ D2(1,j)=C2(1,j)\oplus X(1,j)\oplus C2(1,j-1) \\ D3(1,j)=C3(1,j)\oplus X(1,j)\oplus C3(1,j-1) \\ \end{array}\right. } \end{aligned}$$

Step 5: The first column of three pictures is handled by inverse diffusion.

34 $$\begin{aligned} {\left\{ \begin{array}{ll} D1(i,1)=C1(i,1)\oplus X(i,1)\oplus C1(i-1,1) \\ D2(i,1)=C2(i,1)\oplus X(i,1)\oplus C2(i-1,1) \\ D2(i,1)=C2(i,1)\oplus X(i,1)\oplus C3(i-1,1) \\ \end{array}\right. } \end{aligned}$$

Step 6: For the rest of the pixels, operate on them in a row by

35 $$\begin{aligned} {\left\{ \begin{array}{ll} D1(i,j)=C1(i,j)\oplus X(i,j)\oplus C1(i-1,j)\oplus C1(i,j-1) \\ D2(i,j)=C2(i,j)\oplus X(i,j)\oplus C2(i-1,j)\oplus C2(i,j-1) \\ D2(i,j)=C3(i,j)\oplus X(i,j)\oplus C3(i-1,j)\oplus C3(i,j-1) \\ \end{array}\right. } \end{aligned}$$

Step 7: Three sequences *a*$_1$, *b*$_1$ and *q* are acquired the same as "Image fusion" section. Then, the inverse Arnold transform is carried out by

36 $$\begin{aligned} {\left\{ \begin{array}{ll} t1=Q1(i);Q1(i)=Q1(q(i));Q1(q(i))=t1 \\ t2=Q2(i);Q2(i)=Q2(q(i));Q2(q(i))=t2 \\ t3=Q3(i);Q3(i)=Q3(q(i));Q3(q(i))=t3 \\ \end{array}\right. } \end{aligned}$$

three vectors of three images pixels are obtained and shaped into matrixes which include *Q*$_1$, *Q*$_2$, *Q*$_3$.

Step 8: The inverse operation of step two in "Decryption scheme" section follows in

37 $$\begin{aligned} {\left\{ \begin{array}{ll} Q3=Q3\oplus Z \\ Q2=Q2\oplus Q3\oplus Y \\ Q1=Q1\oplus Q2\oplus X \\ \end{array}\right. } \end{aligned}$$

at this moment, the decrypted images including *Q*$_1$, *Q*$_2$ and *Q*$_3$ are acquired.

## Performance analysis

### Simulations results

**Figure 10:**
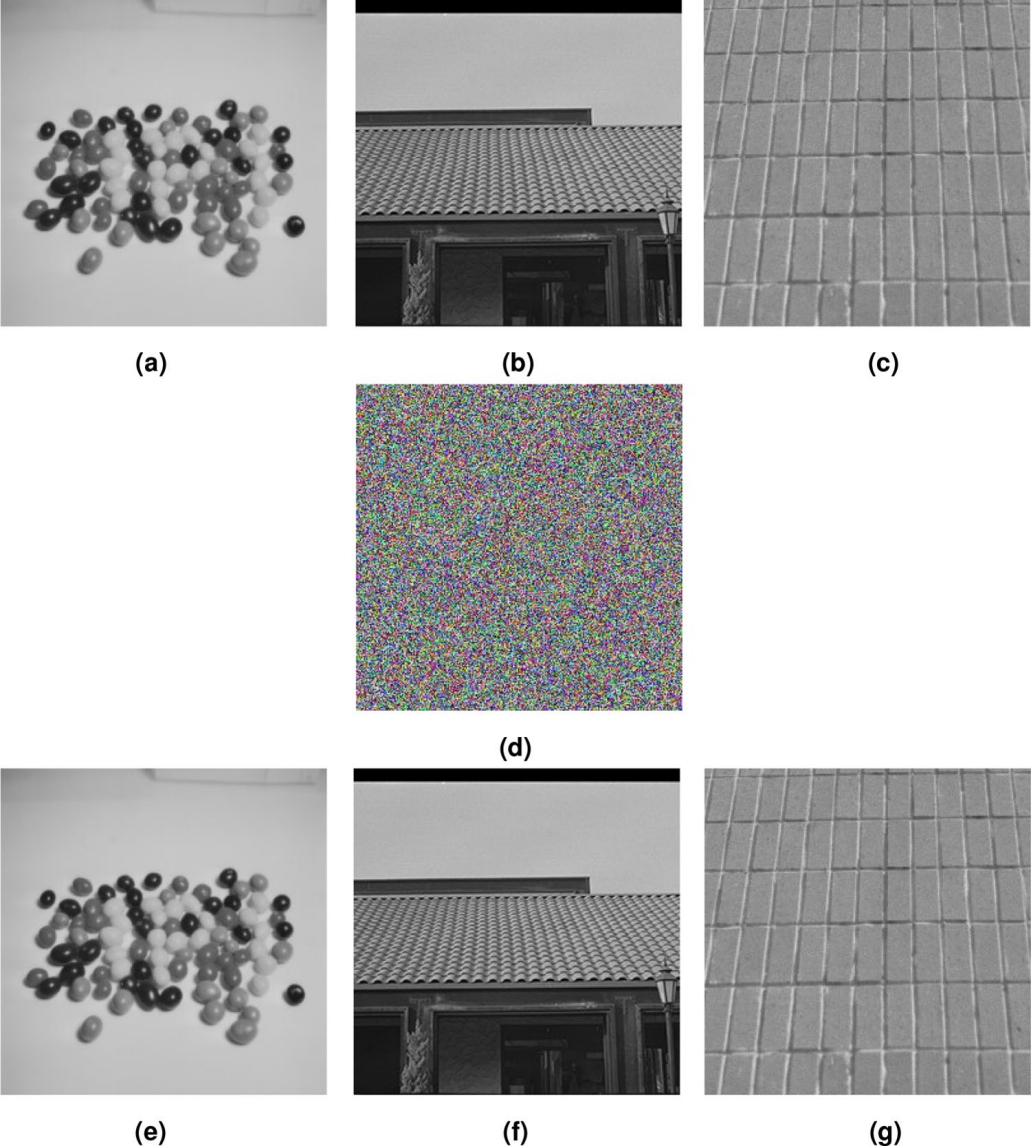
Encrypted and decrypted results, (**a**) OI, Candy, (**b**) OI, House, (**c**) OI, Texture, (**d**) CI, (**e**) DI, Candy, (**f**) DI, House, (**g**) DI, Texture.

To verify the effectiveness of the presented encryption algorithm, the designed image encryption scheme is tested. Deploying step size *h* = 0.01, *c* = 20, *e* = 150/7, *g* = 15, *n* = 0.15, *p* = 3, *s* = 0.05, *m*$_1$ = *m*$_2$ = 0.1, *q* = 0.97, starting value is [*x* *y* *z* *w* *u*] = [0.1 0 0 0 0]. Original image Candy, House and Texture in size 256–256 are encrypted and decrypted simultaneously. The simulation results of proposed image encryption and decryption algorithm are shown in Fig. 10. Where original images (OI) are Fig. 10a–c, cipher image (CI) is displayed in Fig. 10d, the corresponding decryption images (DI) are Fig. 10e–g. As we can see from Fig. 10, the cipher image is visually completely different from plaintext images. The cipher image is almost noisy and is in color. Therefore, the proposed algorithm can encrypt and decrypt images efficiently.

### Key space

The key space of an encryption algorithm should be large enough to resist brute force attacks. This algorithm has fourteen control parameters. The system parameters *c* and *e* change 10$^{-14}$, *g* and *p* change 10$^{-15}$, *n* and *n* change 10$^{-16}$, *m*$_1$, *m*$_2$ and *q* change 10$^{-17}$, the system initial values change 10$^{-17}$. So, the key space of the proposed scheme is more than 2$^{750}$, it is much bigger than 2$^{100}$, which is regarded as the minimum value of key space. Data from other literature are given in Table 2 for reference^53–57^. So, the proposed can stand up to brute force attack.

**Table 2:** Key space of different algorithms.

Our algorithm	Ref.^53^	Ref.^54^	Ref.^55^	Ref.^56^	Ref.^57^
2$^{750}$	2$^{213}$	2$^{580}$	2$^{497}$	2$^{374}$	2$^{399}$

### Key sensitivity

The image cryptosystem has strong sensitivity if the two cipher images have conspicuous difference. On the contrary, the image cryptosystem is insensitive. A well cryptosystem should have high key sensitivity.

**Figure 11:**
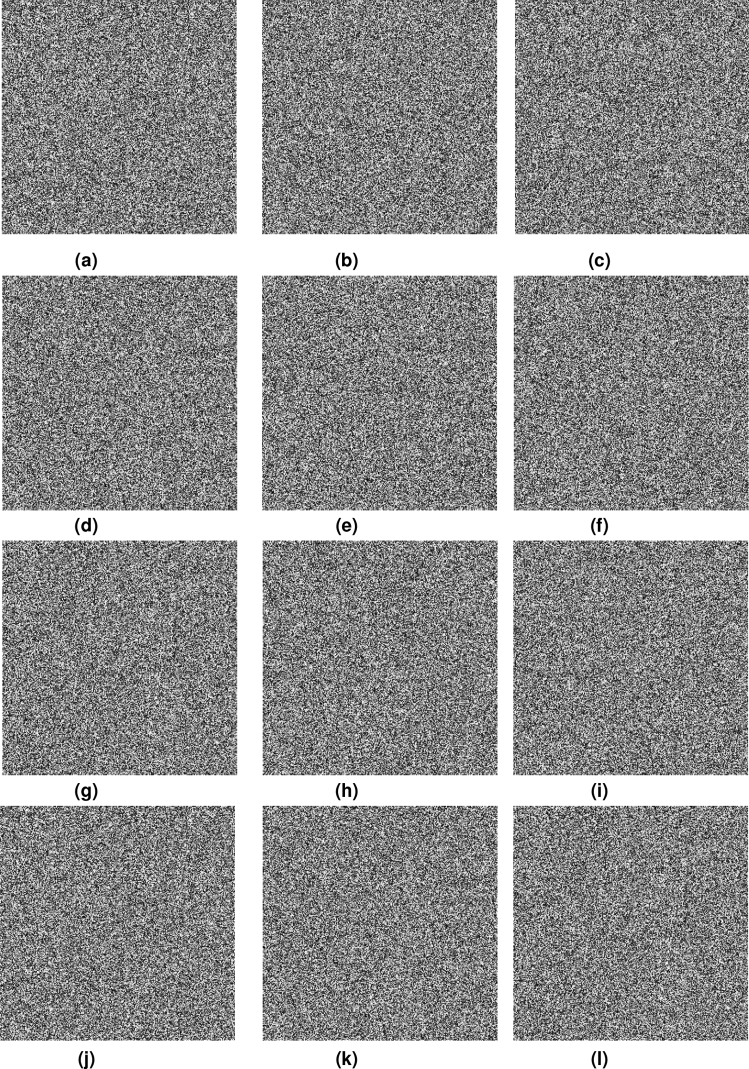
Decrypted results about key sensitivity test, (**a**) Candy, *c* = 20+1$^{-14}$, (**b**) House, *c* = 20+10$^{-14}$, (**c**) Texture, *c* = 20+10$^{-14}$, (**d**) Candy, *g* = 15+10$^{-15}$, (e)House, *g* = 15+10$^{-15}$, (**f**) Texture, *g* = 15+10$^{-15}$, (**g**) Candy, *q* = 0.97+10$^{-16}$, (**h**) House, *q* = 0.97+10$^{-16}$, (**i**) Texture, *q* = 0.97+10$^{-16}$, (**j**) Candy, *m*$_1$ = 0.1+10$^{-17}$, (**k**) House, *m*$_1$ = 0.1+10$^{-17}$, (**l**) Texture, *m*$_1$ = 0.1+10$^{-17}$.

To analyze key sensitivity, the key sensitivity test is done. In the simulation, plain images are encrypted by the slightly altered keys and decrypted by the correct keys. The decrypted images are shown in Fig. 11. Because of the difference in parameter values, sensitivity scales are also different. Via testing one by one, the sensitivity of every parameter can be obtained. From Fig. 11 and the sensitivity of every parameter, the proposed algorithm has highly key sensitivity.

### Histogram

**Figure 12:**
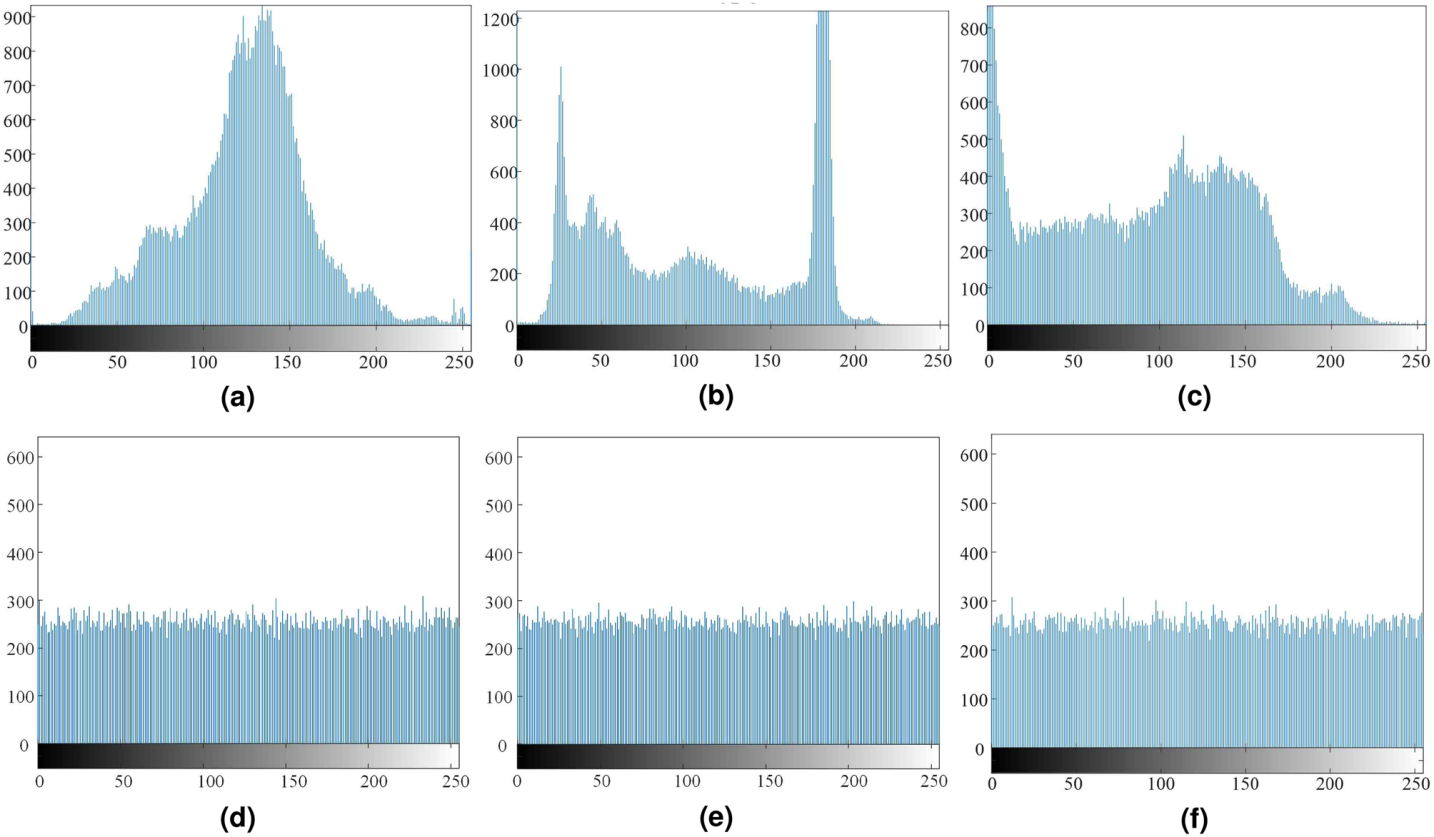
Histogram test, (**a**) OI, Candy, (**b**) OI, House, (**c**) OI, Texture, (**d**) CI, Candy, (**e**) CI, House, (**f**) CI, Texture.

Histogram is a statistic of gray level distribution in gray image. This index can reflect the relationship between the gray level and the frequency. Before encryption, the histogram of the original image is variational. In contrast, the histogram of cipher image is uniform distribution. From Fig. 12, the difference of histogram between original images and cipher images is obvious. The cardinality test can be used to quantitatively analyze the ability of the encryption scheme to resist statistical attacks, and for the cardinality test results are shown in Table 3. The proposed encryption algorithms pass the cardinality test when the significance levels are 0.01, 0.05, and 0.1, respectively. This also shows that the cipher image obtained by the encryption scheme are approximately uniformly distributed^44,58^.

**Table 3:** The table of $\chi ^{2}$-value for different objects.

The model name	$\chi ^{2}$-value (plaintext)	$\chi ^{2}$-value (Cipher)	Critical value		
			$\chi _{0.1}^2$ (255)	$\chi _{0.05}^2$ (255)	$\chi _{0.01}^2$ (255)
Candy (256 $\times$ 256)	49346.0625	243.5469	Pass	Pass	Pass
House (256 $\times$ 256)	72970.4609	228.0781	Pass	Pass	Pass
Texture (256 $\times$ 256)	83678.7734	249.1224	Pass	Pass	Pass

### Correlation of adjacent pixels

Usually, plain images have a strong correlation between adjacent pixels. A good encryption algorithm should generate cipher images with low correlation. In this way, the encryption scheme can hide the original image information. The correlation of adjacent pixels is defined by:

38 $$\begin{aligned}&r_{x,y}=\frac{E((x-E(x))(y-E(y)))}{\sqrt{D(x)D(y)}} \end{aligned}$$

39 $$\begin{aligned}&E(x)=\frac{1}{N}\sum _{i=1}^Nx_i \end{aligned}$$

40 $$\begin{aligned}&D(x)=\frac{1}{N}\sum _{i=1}^N(x_i-E(x))^2 \end{aligned}$$

where E(x) and D(x) are the expectation and variance of the variable *x*, *y*, *r*$_x,y$ is the correlation coefficient between adjacent pixels *x* and *y*.

**Figure 13:**
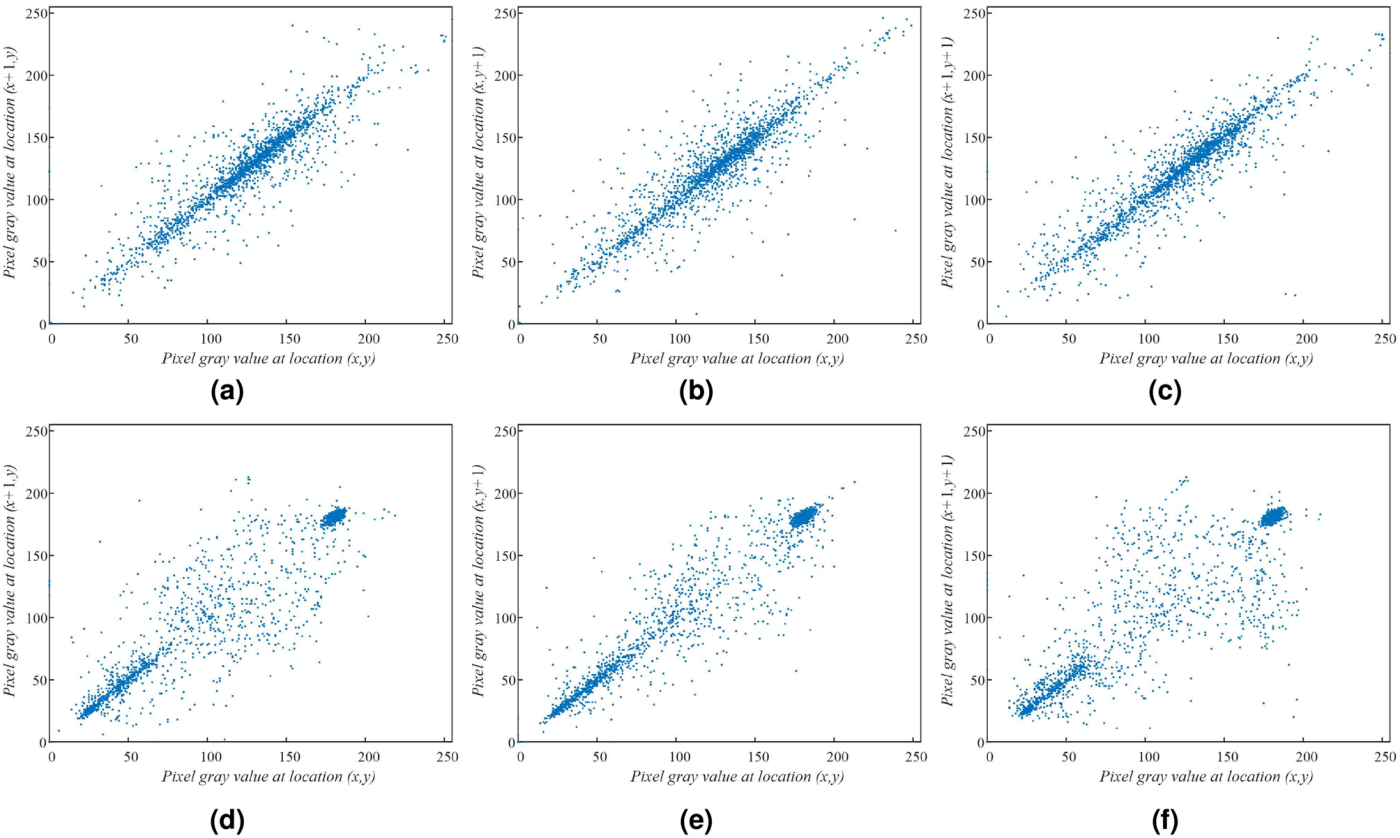
Correlation of adjacent pixels, (**a**–**c**) OI, Candy, (**d**–**f**) OI, House.

**Figure 14:**
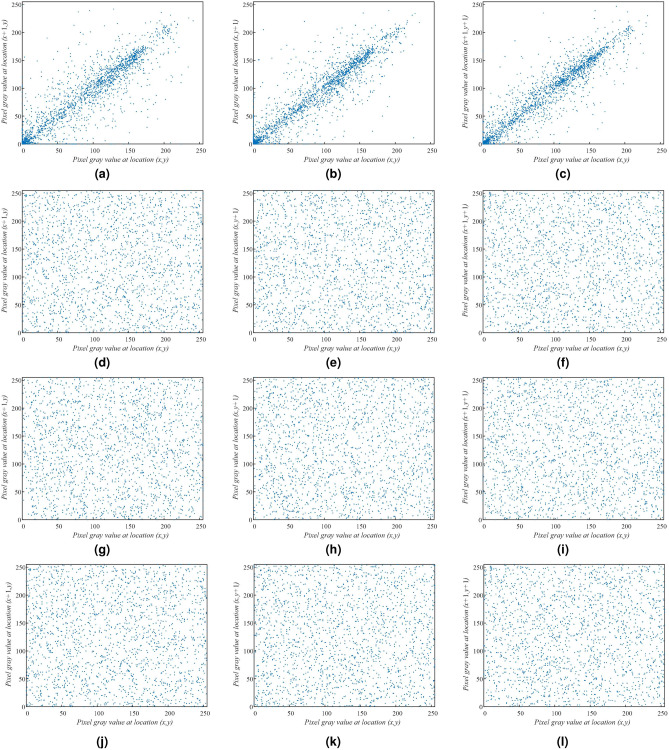
Correlation of adjacent pixels, (**a**–**c**) OI, Texture, (**d**–**f**) CI, Candy, (**g**–**i**) CI, House, (**j**–**l**) CI, Texture.

For testing the correlation of adjacent pixels, we select 1000 pairs adjacent pixels randomly from original images and their corresponding cipher images to analyze. The correlation and correlation coefficients calculated by using the Eq. (38) are shown in Figs. 13 and 14 and Table 4. Results from other literature are also listed in Table 4^59–61^. From Figs. 13 and 14, the adjacent values of plain image pixels all lie near a straight line with slope 1, there is a high correlation between two adjacent pixels. The pixel values of cipher images are carpeted with the whole region, that is to say a low correlation between adjacent pixels. The results in Table 4 also indicate that the correlation coefficients between the adjacent pixels of the original images in horizontal, vertical and diagonal (H, V and D) directions are large. The correlation coefficients of the encrypted image in corresponding orientations are decreased significantly. The encryption algorithm proposed can effectively against statistical attacks.

**Table 4:** Correlation coefficient pixels.

Encryption algorithm	Image	Direction	Plain image	Cipher image
Our scheme	Candy	H	0.9718	0.0005
		V	0.9743	0.0009
		D	0.9506	0.0018
	House	H	0.9254	0.0009
		V	0.9083	0.0036
		D	0.9314	0.0042
	Texture	H	0.9706	0.0045
		V	0.9503	0.0028
		D	0.9465	0.0010
Ref.^60^	Image	H	0.9724	0.0118
		V	0.9455	−0.0173
		D	0.9214	0.0080
Ref.^59^	Image	H	0.9724	−0.0048
		V	0.9455	−0.0112
		D	0.9214	−0.0125
Ref.^61^	Image	H	0.9724	0.0070
		V	0.9455	−0.0102
		D	0.9214	0.0030

###  Information entropy

Information entropy can be used to describe the uncertainty of picture information and to measure its randomness. For an image, the more homogeneous the gray values distribute, the bigger the information entropy is. The picture information has a strong randomness when the information entropy is close to 8. Information entropy is computed by:

41 $$\begin{aligned} H(m)=-\sum _{i=1}^{255}P(x_i)log_2P(x_i) \end{aligned}$$

where P(*x*$_i$) is the probability of gray value *x*$_i$.

Information entropies of original images and cipher images are listed in Table 5. The information entropies of cipher images are more than 7.997 and close to 8. From Table 5, the information entropy of our scheme and others in Refs.^33,41,60,62^ are given, a conclusion that the proposed algorithm can generate cipher images with strong randomness can be drawn.

**Table 5:** Information entropy of original images and cipher images.

Encryption algorithm	Image	Image size	Original image	Cipher image
Our scheme	Candy	256256	7.3456	7.9973
	House	256256	7.1235	7.9975
	Texture	256256	7.0384	7.9976
Ref.^60^	Airplane	256256	–	7.9971
Ref.^62^	Baboon	256256	7.1273	7.9974
Ref.^62^	Average	256256	7.4127	7.9973
Ref.^33^	Average	256256	7.3446	7.9970
Ref.^41^	Average	256256	7.6560	7.9969

### Differential attack

The performance of anti-differential attack depends on the sensitivity to plaintext and is usually measured by the number of pixels change rate (NPCR) and the unified average changing intensity (UACI). NPCR and UACI are calculated by:

42 $$\begin{aligned}&NPCR(P_1,P_2)=\frac{1}{MN}\sum _{i=1}^M\sum _{j=1}^N\mid Sign(P_1(i,j)-P_2(i,j)) \mid \times 100\% \end{aligned}$$

43 $$\begin{aligned}&UACI(P_1,P_2)=\frac{1}{MN}\sum _{i=1}^M\sum _{j=1}^N \frac{\mid P_1(i,j)-P_2(i,j)\mid }{255-0} \times 100\% \end{aligned}$$

where *P*$_1$ on behalf of cipher image and *P*$_2$ is the cipher image which plain image pixel value has changed.

Due to the arbitrariness of position, the theoretical values of NPCR and UACI are 99.6094% and 33.4635% respectively. The NPCR and UACI values in the simulation test should be close to expectation. Via simulation test, the results of the proposed algorithm are presented as Table 6. From the Table 6, the results are closed to theoretical expectations and it will get an almost completely different image if the gray value of the image is changed slightly. Moreover, we list the average values of NPCR and UACI in other literature which is shown in Table 7^10,17,27,63^. Results indicate that our algorithm can resist differential attack effectively.

**Table 6:** The results of differential attack test.

Image	Candy	House	Texture	Average
NPCR (%)	99.5986	99.6232	99.5853	99.6024
UACI (%)	33.5052	33.4633	33.5240	33.4975

**Table 7:** NPCR and UACI values of different algorithms.

	Our algorithm	Ref.^10^	Ref.^17^	Ref.^63^	Ref.^27^
NPCR (%) (average)	99.6024	99.610	99.6117	99.6082	99.5582
UACI (%) (average)	33.4975	33.462	33.6694	33.3391	33.3844

### Robustness

When transmitted over a channel, the cipher image will be influenced by a variety of interference and attacks. A good encryption algorithm should make images have robustness for external interference. Noise attack and cropping attack testing experiments were carried out to test the robustness of the encryption algorithm.

#### Noise attack

**Figure 15:**
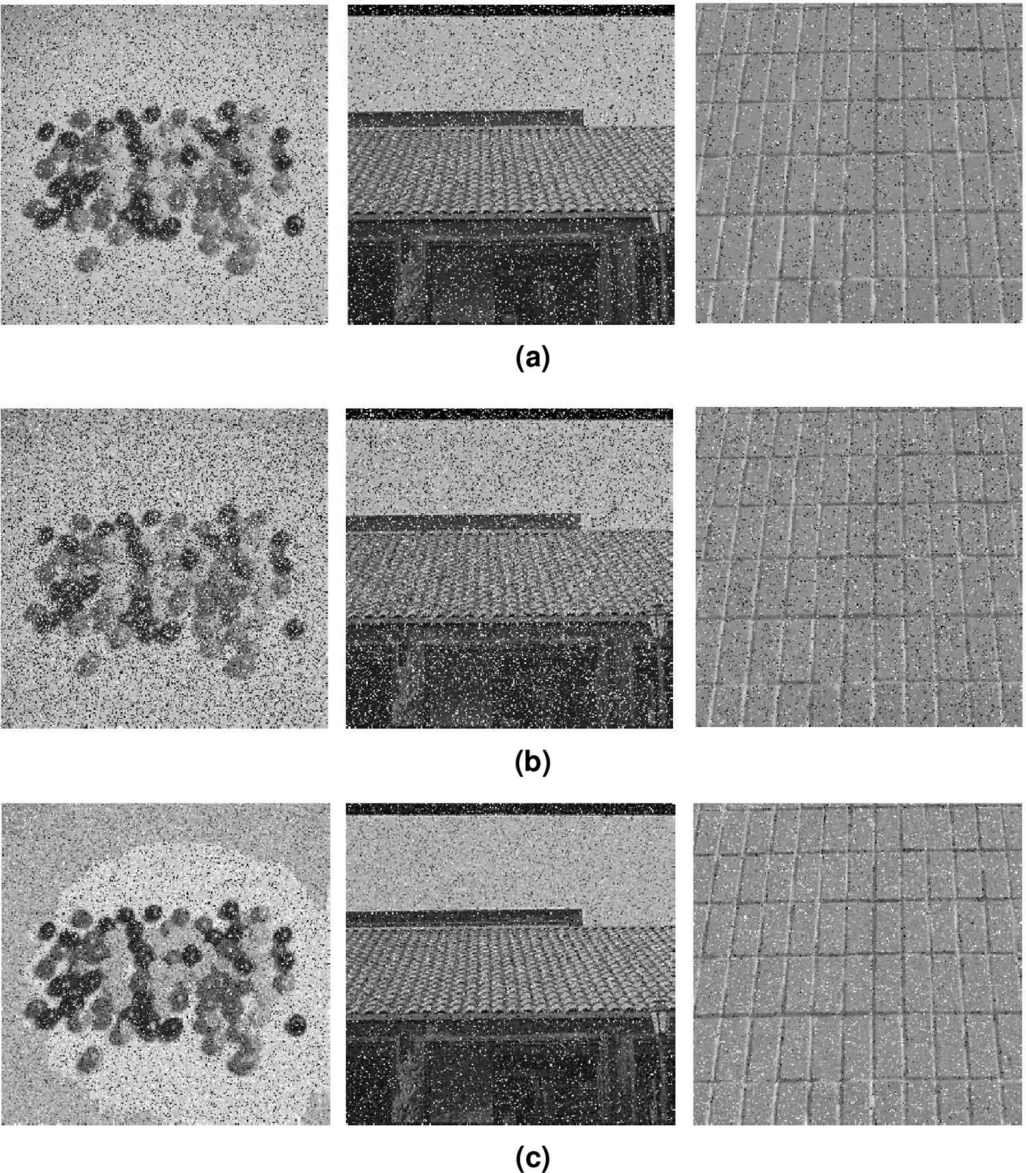
Decrypted images with various noise, (**a**) SPN, 0.05, (**b**) SPN, 0.07, (**c**) GN, 0.0001.

In the process of data transmission, cipher image will be contaminated by noise. For testing the resistance performance of encryption algorithm to noise, Salt and Pepper noise (SPN), Gaussian noise (GN) are added to the cipher image and the decrypted results are shown in Fig. 15. It is observed that the decrypted images still have noise, but the main information can be recovered. So, a certain level of noise attack can be tolerated by the encryption algorithm.

#### Cropping attack

**Figure 16:**
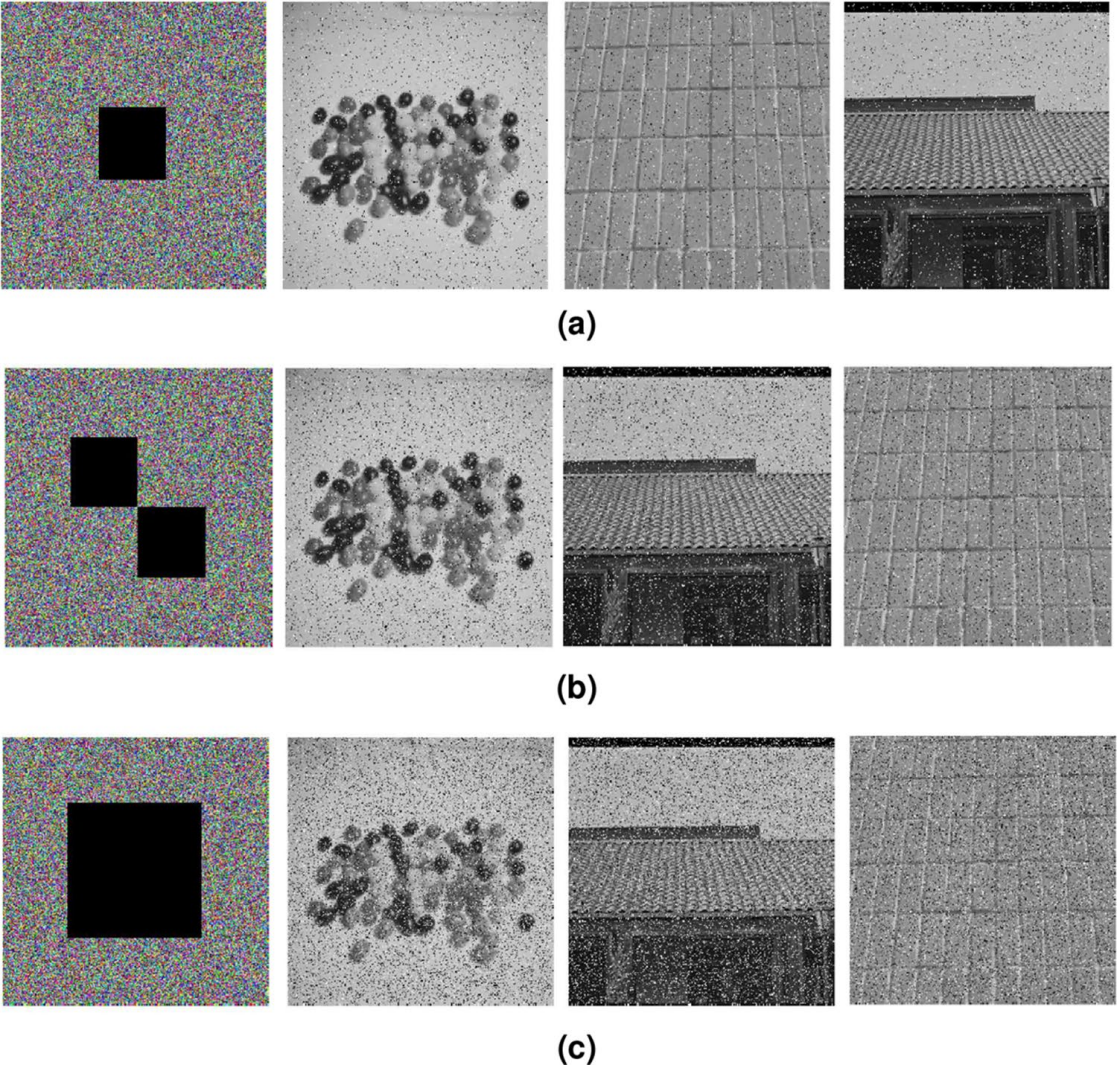
Cropping attack test, (**a**) 6.25% data loss, encrypted image and decrypted images, (**b**) 12.5% data loss, encrypted image and decrypted images, (**c**) 25% data loss, encrypted image and decrypted images.

Cipher image may be destroyed while it is in the process of transmission and results in data loss. The cropping attack test is carried out to illustrate the performance of the proposed encryption algorithm to resist cropping attack. The simulation results are shown in Fig. 16, while encrypted image lose 6.25% data, decrypted images which include Candy, House and Texture are Figure 16a. While encrypted image 12.5% data are cropped, decrypted images are shown in Fig. 16b. While encrypted image 25% data are removed, the results of decryption are shown in Fig. 16c. We can see that though the encrypted image loses 6.25%, 12.5% or 25% data, the main information in the decrypted images can still be identified. Simulation results demonstrate that the proposed algorithm has a certain ability to resist cropping attack.

### Time analysis

Time complexity is an important aspect to measure the efficiency of the encryption algorithm,for three images ‘Candy’, ‘House’ and ‘Texture’, the running time for encryption and decryption is shown in Table 8 and compared with other encryption schemes as shown in Table 9. From the Table 9, it can be seen that the encryption scheme has a better performance in terms of running rate^47,64–67^.

**Table 8:** Running time of the proposed algorithm.

Round	1 (s)	2 (s)	3 (s)	4 (s)	5 (s)	6 (s)	Average (s)	Speed (Mb/s)
Encryption	0.0382	0.0404	0.0459	0.0441	0.0467	0.0426	0.0430	4.4967
Decryption	0.0326	0.0272	0.0360	0.0366	0.0602	0.0464	0.0399	4.8461

**Table 9:** Running time of the different algorithm.

Algorithm	Ours	Ref.^47^	Ref.^64^	Ref.^65^
Encryption	0.0430	0.4400	1.1737	0.3356
Decryption	0.0399	–	–	1.5216

## Conclusion

In this paper, a multiple image encryption scheme based on fractional-order hyperchaotic system is presented. The phase diagram, bifurcation diagram, Lyapunov exponent spectrum and equilibrium point are analyzed in detail. The analysis results show that the fractional-order hyperchaotic system has complex dynamical characteristics and it is suitable for image security encryption. The fractional-order hyperchaotic system is implemented on the DSP platform and the results are the same as simulation results. It provides the possibility of realizing secure communication with fractional-order hyperchaotic systems. By using the proposed algorithm, multiple images are encrypted twice, it not only improves the encryption efficiency, but also improves the security of image transmission. The key space, key sensitivity, histogram, correlation, information entropy and robustness are analyzed, the results indicate that it can withstand brute attack, statistical attack, a certain degree of noise pollution and cropping attack effectively. It shows that the encryption algorithm has a great encryption effect. Hence, the proposed image encryption scheme has research significance and application value.

## Data Availability

The test images used in this paper are from the SIPI image database and are used for scientific research only, not for other purposes, and without copyright disputes.
